# Store-specific grocery shopping patterns and their association with objective and perceived retail food environments

**DOI:** 10.1017/S1368980023002720

**Published:** 2023-12-11

**Authors:** Daisy Recchia, Marlène Perignon, Pascaline Rollet, Nicolas Bricas, Simon Vonthron, Coline Perrin, Lucie Sirieix, Hélène Charreire, Caroline Méjean, Surfood-Foodscapes Working Group

**Affiliations:** 1 MoISA, Univ Montpellier, CIRAD, CIHEAM-IAMM, INRAE, Institut Agro, IRD, Montpellier, Occitanie, France; 2 CIRAD, UMR MoISA, F-34398 Montpellier, Occitanie, France; 3 INNOVATION, Univ Montpellier, CIRAD, INRAE, Institut Agro, Montpellier, Occitanie, France

**Keywords:** Food purchasing, Food supply sources, Retail food outlets, Store choice, Food environment, Activity space

## Abstract

**Objective:**

To explore store-specific grocery shopping patterns and assess associations with the objective and perceived retail food environment (RFE).

**Design:**

This cross-sectional study used principal component analysis and hierarchical cluster analysis to identify grocery shopping patterns and logistic regression models to assess their associations with the RFE, while adjusting for household characteristics.

**Setting:**

The Montpellier Metropolitan Area, France.

**Participants:**

To be eligible for inclusion, participants had to be 18 years of age or older and reside in the Montpellier Metropolitan Area. Analyses were carried out on 415 households.

**Results:**

Households of cluster ‘Supermarket’ (49 % of households) primarily shopped at supermarkets and were less likely to live near a convenience store. Households of cluster ‘Diversified’ (18 %) shopped mostly at organic stores, at markets, at specialised stores, and from producers and were more likely to have a market in their activity space. Households of cluster ‘Discount’ (12 %) primarily shopped at discounters and were less likely to perceive a producer in their activity space. Households of cluster ‘Convenience’ (12 %) mostly shopped online or in convenience stores. Finally, households of cluster ‘Specialized’ (9 %) had high expenditures in greengrocers and in other specialised food stores and were more likely to live near a specialised food store.

**Conclusions:**

This study highlighted the importance of considering both perceived and objective RFE indicators, as well as assessments around the home and in activity space. Understanding how people buy food and interact with their RFE is crucial for policymakers seeking to improve urban food policies.

Obesity rates have risen globally, leading researchers to investigate factors that may influence them^(1)^. The food environment (FE), which encompasses the interface between consumers and the food system, including the availability, affordability, convenience, quality and promotion of foods^(2)^, has been studied as a potential factor related to weight status in numerous studies^(3,4)^. The retail food environment (RFE) or ‘foodscape’ is a dimension of the FE^(5)^, that refers to the physical, sociocultural and economic space in which individuals encounter food and meals^(6)^. The results of the associations between the RFE and obesity are often mixed or inconclusive^(4)^.

Although it may seem evident that the RFE initially influences grocery shopping practices, then eating behaviours and finally weight status, grocery shopping practices and, more specifically, the use of certain food supply sources (FSS) are rarely assessed or considered as potential mediating factors related to the RFE^(7)^. In addition, the RFE is often limited to the surrounding residential areas, neglecting the influence of non-residential places of activity and travel behaviours^(8)^. Consumers, however, do not necessarily shop at food outlets that are closest to their home^(9–12)^; frequently visited locations (e.g. workplace) are other areas of exposure to consider^(13)^. Furthermore, the perceived RFE, which refers to consumers’ experience of the RFE, is often overlooked, even though perceived measures of availability have more consistently been associated with dietary outcomes than objective measures^(14)^.

Understanding where groceries are purchased and by whom, in relation to contextual factors, is important for several reasons, with significant implications for public health. Such studies shed light on socio-economic and spatial disparities in food access, particularly concerning healthy foods such as fruits and vegetables^(15)^. They, therefore, provide essential information for designing targeted policy interventions to reduce social and spatial inequalities in food behaviours. Ultimately, such research contributes to the broader goal of promoting healthier eating habits, reducing health inequities and improving public health outcomes.

Food shopping practices are deeply linked with consumers’ food culture, which is why analyses in different geographical settings are essential^(16,17)^. France has a particularly diverse range of food outlets, which provides French consumers with many choices when looking for a place to shop for food. French households frequently shop at small specialist shops such as greengrocers, bakeries, butchers, fishmongers and markets, which are, for instance, more abundant in the French RFE compared with central England’s RFE^(18)^. Assessing grocery shopping practices and their associations with the RFE becomes more interesting nowadays, with the emergence of new alternative store formats^(19)^, such as short supply chain distributions and e-commerce, which have become increasingly popular over the last few years.

In this paper, we aimed to explore grocery shopping practices of households in southern France and assess the associations with multiple determinants at individual and contextual levels. To achieve this, we identified grocery shopping patterns based on the share of expenditures by FSS, which we characterised in terms of household characteristics. Then we investigated the associations between the identified patterns and the perceived and objective RFE, independently of household characteristics.

This is the first study that explores the intricate relationship between food purchasing behaviours, summarised as shopper profiles, and the RFE across different scales. Notably, the RFE was evaluated both around the home and within households’ activity space, encompassing areas around places of primary activity and commuting routes.

## Methods

### Study population

The Mont’Panier cross-sectional study was conducted from May 2018 to December 2019. Participants were recruited based on a call for participation, which involved reaching out via various channels such as local newspapers, radio programmes, interviews on regional television news channels, as well as the distribution of posters and flyers. The target of this recruitment was volunteer households residing in the Montpellier Metropolitan Area (MMA), which comprises the city of Montpellier and thirty-nine municipalities surrounding it. To be eligible for inclusion in the study, participants had to be 18 years of age or older and participate at least partly in grocery shopping. The survey involved two data collection tools: an online questionnaire and a food supply diary. The questionnaire gathered information on the household’s socio-economic and demographic (SED) characteristics, frequent activity locations and transportation methods, perception of the RFE and food purchasing behaviours. The main respondent answered these questions even if they concerned other household members. The household head was defined as the oldest active adult (or the oldest adult if there was no active member in the household), based on the definition used by the French institute of statistics (INSEE). The food supply diary was used to collect detailed information on food and beverage purchases for at-home consumption made during 1 month, including location of purchase and details of the items purchased (e.g. price). Participants were encouraged to provide receipts whenever possible.

Quota sampling was performed based on household composition (single adult, multiple adults, single adult with at least one child and multiple adults with at least one child) crossed with the age group of the household head (<30, 30–50 and >50 years), using sociodemographic data of the MMA from the INSEE.

### Grocery shopping practices

Households’ food purchases were evaluated over a 1-month period using food supply diaries and grocery receipts. Only food purchases for at-home consumption were considered for this study. To impute missing data regarding expenses for food items (*n* 454/58711; 0·77 %), we employed the INSEE database and food store websites. Mean prices per kilogram were multiplied by the quantity (in kilograms) when available. In cases where the quantity was missing, the quantity was initially imputed using the mean quantity of the respective food item within the study sample. The share of expenditures by FSS for each household was calculated by dividing the household’s food expenditures at a specific food store by the total food expenditures across all food stores for that household. FSS were classified into eleven categories, namely supermarkets (including hypermarkets), discounters, organic stores, markets (open-air and covered), convenience stores, online shopping, bakeries, greengrocers, specialised stores (butchers, fishmongers and dairy stores), frozen food stores and direct sales from producers. Producers include market gardeners (known as *‘maraîchers’* in French), farm shops, roadside markets, Associations for the Maintenance of Peasant Agriculture (AMAP) – a French version of Community Supported Agriculture (CSA), and basket orders with home delivery or pick up at the farm or drop-off-location.

### Household characteristics

The Mont’Panier study collected SED data through the online questionnaire. Household income per unit of consumption was categorised as follows: <980 €, 980–1722 €, 1723–2550 € and >2550 € per month (quartiles of income from the study population, the MMA). Missing data on income (73/665, 11 %) were imputed through simple linear regression using the following variables: household composition, household head’s age, employment status and education level. Household composition was categorised as one adult, multiple adults, one adult with at least one child and multiple adults with at least one child. Other variables included the household head’s age group (<35, 35–50 and >50 years), level of education (high school degree or lower, undergraduate degree, and postgraduate degree), employment status (employed *v*. not), car ownership (yes *v*. no) and home addresses. Home addresses were categorised into three geographic classes of living locations: districts of the city centre of Montpellier, districts around the city centre of Montpellier, and municipalities surrounding Montpellier.

Identification with suggested buyer profiles was self-reported through responses to questions about participants’ main aim or interest while grocery shopping. The following buyer profiles were suggested: aiming for efficiency, looking for promotions/best prices, thinking about meal preparation, interested in social interactions (enjoying talking to vendors or other customers) and aiming for enjoyment/pleasure. Participants were asked to rate their agreement with each statement on a five-point Likert scale, ranging from strongly agree to strongly disagree. Responses were dichotomised into yes (strongly agree and agree) or no.

In addition, the online questionnaire included questions about participants’ satisfaction with the RFE and requested improvements concerning the accessibility of supermarkets, markets, proximity stores, cheaper foods and online food shopping opportunities.

### Perceived retail food environment

The perceived RFE was assessed using questions from the online questionnaire concerning perceived availability (yes *v*. no) of FSS in activity space (around the home and regularly frequented places by household members who participate in grocery shopping). The considered FSS were supermarkets (including hypermarkets), discounters, organic stores, markets, convenience stores, greengrocers, specialised food stores, frozen food stores and producers (as defined above).

### Objective retail food environment

The location of FSS was obtained through the Système d’Identification du Répertoire des Entreprises et de leurs Établissements (SIRENE) database of January 2019. SIRENE is a national business and establishment register database managed by the INSEE that records the identity of all active companies and their establishments in France. This database was first cross-checked using the database of OpenStreetMaps from April 2018, which provides open data of companies and establishments that can be updated and enriched by external contributors. Later, online searches on Google Maps, company websites of major food retailers and city websites (e.g. for information about local markets) were performed to further verify the database. Final verifications were done through field observations of about 5 % of the studied area: the information provided on the location and type of FSS by the database were validated through ground-truthing in the city region of Montpellier between May 2018 and January 2019^(20)^.

The classification of FSS was based on the initial classification of food stores in the SIRENE database. This study focused on the following types of FSS for the objective RFE: supermarkets, e-supermarkets (online food purchasing with pickup at supermarkets, known as ‘drive’ in French), organic stores, markets (open-air and covered), convenience stores, bakeries, greengrocers and other specialised food stores (butchers, fishmongers and dairy stores).

The objective RFE was assessed around participants’ home and in their activity space. Addresses of their current home and main places of activity (i.e. work and/or other places they reported visiting at least once a week) were collected through the online questionnaire. Main places of activity of all household members who reported participating in grocery shopping, even if their involvement was minimal, were considered to determine the household’s activity space. The question concerning main places of activity was phrased as follows: ‘We need to know the address of the place(s) you regularly frequent in order to determine the scope of your FE. Is there a place (other than your home) that you visit regularly (at least once a week)? Examples include school, daycare, leisure activity location (such as sports or music), the residence of a family member’. In addition, the main mode of transportation (e.g. walking, biking, and car) used to and from the reported activities was collected. All addresses were geocoded using QGIS v3.4.7. The objective RFE was assessed using geographic information systems, with the geocoded addresses of the FSS and household members’ home and main places of activity.

Three types of objective RFE indicators were assessed: proximity of FSS to the home, presence of FSS around the home and presence of FSS in household members’ activity space. The activity space was defined as the exposure environment, including areas around the home, around household members’ main places of activity and commuting routes between those places.

The proximity of each FSS was calculated by assessing the Euclidian distance between the nearest FSS (of each type), relative to each home address. Given the non-normal distribution of proximity indicators, normalisation through log +1 transformation was undertaken.

The presence of small food stores (convenience stores, bakeries, greengrocers and other specialised food stores) around the home was assessed using a 500-m road network buffer. Since a substantial proportion of households were lacking access to other types of FSS (supermarkets, e-supermarkets, organic stores and markets) within this range, a larger buffer of 1000 m was employed for these particular FSS. These buffer sizes are commonly used to assess exposure to RFE^(21)^.

The presence of each FSS in households’ activity space was calculated within a 500-m road network distance around households’ home address and other places of activity, as well as 100-m or 300-m along commuting routes between those places. Commuting routes were computed based on the shortest street network distance, and a specific buffer was used depending on the modes of transportation reported in the online questionnaire. Specifically, a 100-m buffer was used for walking and cycling journeys to capture the environmental context experienced by pedestrians and cyclists^(22–24)^, while a 300-m buffer was used for car and motorcycle journeys, based on measurements of the distance between hypermarkets and the nearest primary road axis in our study area.

### Statistical analysis

Analyses were conducted on households with complete data for the variables used in the study, following imputation for missing food expenditure and income data. A weighted sample was used for all analyses presented in this study. Weights were calculated using the ‘icarus’ package of R Statistical Software (version 4.1.0) to ensure that the marginal distribution of the weighted sample was consistent with that of the targeted population. The sample was calibrated based on income per unit of consumption and household composition crossed with the age group of the household head, using data from the 2017 INSEE database.

In order to identify patterns of grocery shopping practices, a two-step approach was employed, starting with a principal component analysis (PCA) followed by a cluster analysis. These analyses were conducted on the shares of expenditures by different FSS. First, PCA was conducted to uncover underlying patterns and dimensions within the data (Additional file 1). The number of retained dimensions was chosen to obtain a cumulative percentage of variance of 95 %^(25)^. The inclusion of PCA was essential in capturing the nuanced relationships among variables and informing subsequent clustering. The results of the PCA were omitted from the manuscript to prevent unnecessary overload of information, they are however available in Additional file 1. A cluster analysis was then performed to identify distinct grocery shopping patterns based on the PCA results. Clustering was conducted using Ward’s hierarchical classification of individuals, followed by K-means clustering, to maximise inter-class inertia. The number of clusters was determined using inter-cluster inertia gain and graphical observation of the dendrogram^(26)^. Cluster analysis yielded groups, labelled according to the FSS with significantly higher share of expenditures, which were interpreted as patterns of grocery shopping practices.

Households’ SED characteristics, mean share of expenditures by FSS as well as reported identification with suggested buyer profiles, satisfaction with the RFE, and requested improvements concerning the RFE were described for the total sample and for each cluster individually. Descriptive statistics were expressed as weighted percentages and means and standard deviation. Chi-squared tests with Rao & Scott’s second-order correction were used for categorical variables, and Wilcoxon rank-sum tests for complex survey samples were used for numerical variables.

To determine the strength of the associations between each cluster membership and each explanatory variable (RFE indicators), binary logistic regression models were performed, calculating Odds Ratio (OR) and 95 % Confidence Interval (CI). The rationale behind using logistic regression was to determine whether the RFE served as a determinant of the identified patterns. Notably, the RFE variables were not explored within the cluster analysis in order to study how explanatory variables were associated with the identified clusters. Separate models were conducted for each cluster, comparing households within each cluster to all other households in the study sample. Only explanatory variables associated with clusters at a significance level of *P* < 0·1 in bivariate analyses were retained for inclusion in the subsequent multivariate models. A multivariable backward-stepwise logistic regression was performed to determine the variables included in the final models, with income per unit of consumption, household composition and age of household head forced into the model. Variables whose exclusion from the model caused large fluctuations in OR (>10 %), as well as variables whose exclusion increased the significance of the likelihood ratio tests (*P* > 0·05), were re-entered into the model.

Four separate multivariate models were performed. The first two models assessed associations with the objective RFE assessed around the home, with the first model including proximity indicators and the second model including presence indicators. The third model included objective RFE indicators assessed in activity space, namely presence of FSS, and the fourth model included perceived RFE variables. All four models were adjusted for socio-economic characteristics. Statistical analyses were conducted using R Statistical Software (version 4.1.0), and the threshold for statistical significance was set at *P* < 0·05.

## Results

### Description of the study sample

Out of the 738 participants enrolled in the Mont’Panier study, analyses were conducted on 415 households with complete data for the variables used in this study. Given the sample’s adjustment by calibration on margins based on SED characteristics of the MMA population, distributions correspond to those of the general population. These results are presented in the first column of Table 1.

**Table 1:** Households’ socio-economic and demographic characteristics

	Total sample	Cluster Supermarket 49 %		Cluster Diversified 18 %		Cluster Discount 12 %		Cluster Convenience 12 %		Cluster Specialised 9 %	
	%*	%*	*P* †	%*	*P* †	%*	*P* †	%*	*P* †	%*	*P* †
**Income per unit of consumption**			>0·9		**0·01**		**0·002**		0·8		0·5
<980 Euros/month	25	25		10		**48**		19		31	
980–1722 Euros/month	25	24		28		28		29		17	
1723–2549 Euros/month	25	26		30		14		25		22	
>2550 Euros/month	25	25		**32**		11		28		30	
**Household structure**			0·3		0·3		0·3		**0·08**		**0·05**
One adult without children	44	43		38		54		**47**		38	
One adult with child(ren)	10	13		5		12		5		3	
Multiple adults without children	26	25		32		20		13		**45**	
Multiple adults with child(ren)	20	19		25		14		35		14	
**Age**			0·8		**0·02**		>0·9		0·2		0·5
< 35 years	29	29		19		30		41		31	
35–50 years	26	25		38		27		21		18	
> 50 years	45	46		**43**		43		39		50	
**Education**			0·3		**0·05**		**0·08**		0·3		0·3
High school education or less	23	26		13		31		12		25	
Bachelor’s degree	35	35		33		**42**		40		24	
Master’s degree or more	42	39		**53**		27		48		52	
**Employment status**	70	73	0·4	**80**	**0·07**	50	**0·003**	77	0·3	54	**0·03**
**Car ownership**	83	85	0·5	86	0·4	68	**0·009**	85	0·7	83	>0·9
**Living location**			0·1		0·4		0·4		0·7		0·4
City centre of Montpellier	18	14		24		17		22		26	
Around the city centre of Montpelier	42	43		39		52		37		35	
Municipalities surrounding Montpellier	40	43		38		31		41		39	

* Weighted percentage.

† Chi-squared test with Rao & Scott’s second-order correction.

The sample was adjusted by calibration on margins based on income per unit of consumption and household composition crossed with household head’s age group. The numbers in bold represent percentages higher than those of the total sample, and *P*-values < 0·1.

The first column of Table 2 presents mean share of expenditures by FSS for the total sample. Half of households’ food expenditures seem to have been made in supermarkets, a little more than one-tenth of expenditures were made in discounters and about one-fifth of expenditures were made in alternative FSS, such as organic stores, markets and to a lesser extent producers. Ten per cent of expenditures were made in convenience stores or online, with greater expenditures for convenience stores than for online stores. The remaining expenditures were made in specialised food stores, including bakeries, greengrocers, fishmongers, butchers, dairy stores and frozen food stores.

**Table 2:** Mean share of expenditures by food supply source

	Total sample		Cluster Supermarket 49 %			Cluster Diversified 18 %			Cluster Discount 12 %			Cluster Convenience 12 %			Cluster Specialised 9 %		
	Mean	sd *	Mean	sd *	*P* †	Mean	sd *	*P* †	Mean	sd *	*P* †	Mean	sd *	*P* †	Mean	sd *	*P* †
Supermarket	50·5	31·5	**76·9**	**16·3**	**<0·001**	23·5	17·7	**<0·001**	27·5	20·1	**<0·001**	18·6	18·0	**<0·001**	32·4	18·6	**<0·001**
Discounter	11·2	21·5	5·1	8·8	**0·003**	1·3	4·0	**<0·001**	**60·8**	**22·3**	**<0·001**	3·5	7·7	**0·02**	8·7	12·4	0·7
Organic store	8·3	17·6	3·3	6·8	**<0·001**	**30·9**	**29·1**	**<0·001**	0·9	3·1	**<0·001**	4·9	8·9	0·6	4·3	8·2	0·2
Market	6·9	14·0	3·1	6·6	**<0·001**	**20·6**	**22·9**	**<0·001**	3·3	7·9	**0·012**	3·9	8·3	0·2	8·8	14·9	0·2
Producer‡	3·8	9·1	2·6	6·8	0·2	**11·0**	**15·3**	**<0·001**	0·5	2·5	**<0·001**	1·6	4·0	0·3	2·2	6·2	0·6
Convenience store	6·7	16·7	2·0	5·6	**<0·001**	3·4	8·6	0·4	2·3	5·5	0·5	**37·2**	**31·9**	**<0·001**	5·9	10·0	0·2
Online shopping	3·7	12·2	0·9	3·6	**<0·001**	1·7	5·2	0·6	1·1	5·8	**0·004**	**23·5**	**26·3**	**<0·001**	1·6	6·3	0·6
Bakery	1·9	3·8	1·9	4·1	0·4	**2·4**	**4·0**	**0·01**	0·5	1·7	**<0·001**	2·0	3·5	0·6	2·4	3·8	0·2
Greengrocer	1·8	5·1	0·7	2·0	**<0·001**	0·9	2·4	0·7	0·9	2·5	0·5	0·8	1·9	0·3	**12·3**	**11·7**	**<0·001**
Specialised stores§	2·6	6·7	0·9	2·5	**<0·001**	**2·8**	**4·6**	**0·02**	0·7	2·3	**0·002**	1·3	3·8	0·11	**16·4**	**14·1**	**<0·001**
Frozen food store	1·2	4·0	1·2	4·0	0·9	0·5	2·6	**0·04**	1·0	4·3	0·11	0·8	2·5	0·7	**3·4**	**6·7**	**0·002**

* Standard Deviation.

† Wilcoxon rank-sum test for complex survey samples.

‡ Producer: direct sales from producers (e.g. fruit and vegetable growers (called maraîchers in French), farmers, basket orders from Associations for the Maintenance of Peasant Agriculture (AMAP), which is a French version of Community Supported Agriculture).

§ Specialised stores: include butchers, fishmongers and dairy stores.

The sample was adjusted by calibration on margins based on income per unit of consumption and household composition crossed with household head’s age group. The numbers in bold represent mean share of expenditures higher than those of the total sample, and *P*-values < 0·1.

The first column of Additional file 2 presents the descriptive results of reported identification with suggested buyer profiles. The majority of households reported thinking about the meals they intended to cook while grocery shopping. Efficiency was the main priority for nearly 60 % of households, followed by seeking out promotions. More than half of the total sample reported identifying grocery shopping as a pleasurable activity, while only a few households reported enjoying social interactions while shopping for food.

Most households were satisfied with their FE, but about one-third of households wished for improvements in the availability of markets, one-quarter wished for improvements in access to cheaper foods and one-fifth wished for improvements in the availability of proximity stores. Only a few households requested improvements in online food shopping opportunities and supermarket availability. The results are presented in the first column of Additional file 2.

### Grocery shopping patterns

Five distinct clusters were identified based on households’ share of expenditures across different FSS. Clusters were named after the FSS with significantly higher share of expenditures. SED characteristics and mean share of expenditures for each cluster can be found in Tables 1 and 2, while information on reported identification with suggested buyer profiles, satisfaction with the RFE and requested improvements concerning the RFE by cluster can be found in Additional file 2.

*Cluster ‘Supermarket’* was the largest cluster with 49 % of households of the total sample and comprised households who primarily shopped in supermarkets. No significant difference in SED characteristics was observed between this cluster and others. Households in this cluster were more likely to seek promotions but less likely to enjoy social interactions during grocery shopping. They were also more likely to wish for a better availability of cheaper foods.

The second-largest cluster*, cluster ‘Diversified’* made up 18 % of households and included households who shopped across a range of FSS, with a higher mean share of expenditures in organic stores, markets, and producers, as well as bakeries and specialised food stores compared with other clusters. Households in this cluster were more likely to have higher incomes (>2550 €/month), be older (> 35 years), have a master’s degree or higher, and be employed. They were less likely to prioritise efficiency or seek out promotions, but more likely to enjoy social interactions and experience pleasure while grocery shopping. Households in this cluster were also less likely to wish for improvements concerning the availability of cheaper foods and online food shopping opportunities.

The *‘Discount’ cluster*, accounting for 12 % of households, included households who primarily shopped in discounters. Nearly half of the households in this cluster had low incomes (<980 €/month), and household heads were less likely to have a high education level, be employed, or own a car. Households in this cluster were more likely to seek promotions but less likely to enjoy social interactions during grocery shopping. They were also more likely to wish for improvements concerning access to cheaper food.

The *‘Convenience’ cluster*, also accounting for 12 % of households, comprised households who had a higher share of expenditures in convenience stores and online. The most common household structures in this cluster were single adults and multiple adults with at least one child. Households in this cluster were less likely to seek promotions or enjoy grocery shopping, but they were more likely to enjoy social interactions during the food shopping process.

Finally, the *‘Specialized’ cluster* included 9 % of households of the total sample, which had a higher share of expenditures in greengrocers, other specialised food stores and frozen food stores. This cluster was mostly composed of multiple adults without children and unemployed household heads. Households in this cluster were more likely to experience pleasure while grocery shopping, less likely to wish for improvements concerning the availability of cheaper food, but more likely to request improvements concerning the availability of proximity stores.

It is worth noting that households in the ‘*Supermarket*’ and *‘Discount’ clusters* were less likely to live in the city centre of Montpellier, while households in the *‘Convenience’, ‘Diversified’ and ‘Specialized’ clusters* were more likely to reside there.

### Associations between grocery shopping patterns and the retail food environment

The results of the four multivariate logistic regression models, investigating the associations between grocery shopping patterns and the RFE, are presented in Table 3. To prevent redundancy and reduce the length of the table, only OR and 95 % CI for RFE variables are presented. The variables included in each model are described in the footnote of Table 3. As the results for SED characteristics in the four models are similar to those reported in the preceding paragraph, they will not be repeated here.

**Table 3:** Adjusted multivariate associations between cluster membership and objective, and perceived food environment

	Cluster Supermarket 49 %		Cluster Diversified 18 %		Cluster Discount 12 %		Cluster Convenience 12 %		Cluster Specialized 9 %	
	OR	95 % CI	OR	95 % CI	OR	95 % CI	OR	95 % CI	OR	95 % CI
Model 1. Proximity to home*										
Convenience store	**1·26**	**1·02, 1·56**								
Specialised food stores									**0·63**	**0·45, 0·88**
**Model 2. Presence around the home†**										
Organic food store	0·65	0·38, 1·11								
Convenience store	**0·55**	**0·34, 0·89**			1·81	0·90, 3·64				
Greengrocer									**2·26**	**1·06, 4·82**
**Model 3. Presence in activity space‡**										
E-supermarket	**1·99**	**1·26, 3·14**			0·53	0·27, 1·05			0·55	0·25, 1·21
Market			**1·97**	**1·05, 3·69**						
Bakery	2·01	0·70, 5·76								
**Model 4. Perceived availability§**										
Specialised food store	**0·47**	**0·28, 0·81**								
Food market			1·87	0·93, 3·78						
Producer			**2·32**	**1·34, 4·03**	**0·41**	**0·18, 0·93**				
Frozen food store					0·52	0·25, 1·07				
Discounters									0·58	0·29, 1·18

* **Model 1. Proximity to home:** The proximity corresponds to the shortest distance between the nearest food supply source and each home address; logarithmic transformation was undertaken in order to normalise the variable. Model 1 was additionally adjusted for education level for *cluster* ‘*Diversified’*, and for employment status for *clusters* ‘*Discount’* and ‘*Specialized’*.

† **Model 2. Presence around the home:** The presence of food supply sources around the home was calculated within a 500-m or 1000-m road network distance around each home address. Model 2 was additionally adjusted for education level for *cluster* ‘*Diversified’* and for employment status for *clusters* ‘*Discount’* and ‘*Specialized’*.

‡ **Model 3. Presence in activity space:** The presence of food supply sources in activity space was calculated within a 500-m road network distance around the home and other places of activity, as well as 100 m or 300 m along commuting routes between those places. Model 3 was additionally adjusted for education level for *cluster ‘Diversified’* and for employment status for *cluster ‘Specialized’*.

§ **Model 4. Perceived availability:** The perceived availability was assessed around the home and regularly frequented places by household members who participate in grocery shopping. Model 4 was additionally adjusted for employment status for *cluster* ‘*Specialized’*.

The sample was adjusted by calibration on margins based on income and household composition crossed with household head’s age group. Income per unit of consumption, household structure and age of household head were forced into each of the models. Multivariable backward-stepwise logistic regressions were performed to determine the potential additional adjustment variables included in the final models. The numbers in bold represent results for *P*-values < 0·05.

In the first model, examining the associations with the proximity of FSS to the home, a significant positive association was found between *cluster ‘Supermarket’* and the proximity of a convenience store to the home. This implies that households in this cluster were more likely to live further away from a convenience store than households in the other clusters. Additionally, a significant negative association was observed between *cluster ‘Specialized’* and the proximity of a specialised food store, indicating that households in this cluster were more likely to live close to a specialised food store compared with other clusters.

The second model investigated the associations with the presence of FSS around the home and revealed that households in *cluster ‘Supermarket’* were less likely to be exposed to the presence of a convenience store around their home. On the other hand, households in *cluster ‘Specialized’* were more likely to be exposed to the presence of a greengrocer around their home compared with other clusters.

In the third model, examining the associations with the presence of FSS in households’ activity space, households in *cluster ‘Supermarket’* were more likely to have an e-supermarket in their activity space compared with the four other clusters. Furthermore, households in *cluster ‘Diversified’* were more likely to have a market in their activity space compared with other clusters.

The fourth model investigated the associations with the perceived availability of FSS. Households in *cluster ‘Supermarket’* were less likely to perceive the availability of specialised food stores. However, households in *cluster ‘Diversified’* were more likely to perceive the availability of a producer, while households in *cluster ‘Discount’* were less likely to perceive the availability of a producer in their activity space compared with households in other clusters.

## Discussion

Our study sheds light on the diverse grocery shopping practices of French households and their associations with socio-economic characteristics and consumer profiles, as well as perceived and objective characteristics of the RFE. Our findings show that RFE indicators assessed around the home are especially important when considering proximity stores, while RFE indicators assessed in households’ activity space capture associations, which are often missed when exposure is limited to the residential area. Perceived RFE indicators also play a role in households’ food shopping practices by accounting for their interest in a given FSS.

Concerning consumer profiles, households patronising diversified FSS are more likely to enjoy doing their groceries, whereas one-stop-shop food shoppers are more interested in promotions. As expected, higher-income households are more likely to shop at alternative FSS, such as organic stores^(27)^, markets^(28)^ and CSA^(29,30)^, while lower-income households tend to shop at supermarkets and discounters, which are often considered to provide more affordable foods. The influence of socio-economic status on food shopping decisions revealed potential social disparities. Specifically, households with lower incomes, lower education levels and unemployment are more likely to predominantly patronise discounters. As shown in other studies, socio-economic disparities significantly impact diet quality, with lower-income households often residing in areas dense with discount stores that lack healthier food options^(10,31)^, resulting in poorer diet quality^(32,33)^. These households tend to prioritise promotional offers and cheaper food alternatives, potentially at the expense of healthier food options^(34)^. Conversely, higher-income households have the means to prioritise more pleasurable food purchasing experiences and potentially also healthier food practices^(35)^. The presence of food insecurity thus carries substantial implications for food shopping behaviours, illuminating some underlying factors that drive consumer choices, notably affordability. Interventions in supermarkets^(36)^, farmer’s markets and small food stores which offer diverse, affordable, and healthy food choices have the potential to enhance the RFE and promote healthier eating^(37)^, thus addressing disparities and improving health indicators^(38)^. Therefore, addressing health disparities through the RFE is crucial for public health^(39)^.

Our results show that, while shopping for food, most households primarily think about the meals they intend to cook, and more than half aim to be efficient, find promotions, or enjoy the grocery shopping process. Social interactions are less important, indicating that functional shopping practices are more common than hedonic ones, as identified in another study^(40,41)^. The majority of households in our sample reported being satisfied with their FE, although some expressed a desire for improved availability of markets, while very few wished for more supermarkets in their FE. A study conducted in Montpellier, France, explained that the growing interest in farmers’ markets is partly due to the desire to move away from industrial food production and the French shopper’s appeal to rural heritage and culinary traditions^(42)^. Despite the rich diversity of food outlet types in France, supermarkets remain the most popular FSS for households in our sample, with nearly half of households belonging to the *‘Supermarket’ cluster*. Even households in other clusters have a relatively high mean share of expenditures for supermarkets, and only a modest percentage of households have a higher share of expenditures in alternative FSS such as organic stores, markets or producers.

### Under one-roof grocery shopping practices

Two out of the five identified clusters can be considered *under one-roof* grocery shopping practices, namely the *‘Supermarket’* and *‘Discount’ clusters*. The primary similarity between these clusters is an interest in promotions and cheaper foods, while social interactions during grocery shopping are less likely to be important. These clusters are more likely to correspond to the utilitarian profile, where pleasure is not a sought-after aspect of grocery shopping^(40)^. The two clusters differ mostly by households’ SED characteristics; indeed, households of *cluster ‘Discount’* are more likely to have lower incomes, lower education level, be unemployed and not own a car.

Multivariate analysis showed that households in the *‘Supermarket’ cluster*, compared with households in other clusters, were more likely to live further away from a convenience store, have an e-supermarket in their activity space and not perceive the availability of specialised food stores in their activity space. These results make sense, especially knowing that the vast majority of e-supermarkets in France are also stationary supermarkets. The perceived absence of specialised food stores is logically linked to *under one-roof* food shopping, and the absence of a convenience store in households living environment nudges the consumer to choose supermarkets as their primary, if not exclusive, FSS. Households of *cluster ‘Discount’* did not perceive the availability of a producer in their activity space, which may be due to a lack of interest in these types of FSS, particularly among households with lower incomes who may consider direct sales from farmers too expensive. Although not significantly different from other clusters, households in both the *‘Supermarket’* and *‘Discount’ clusters* were less likely to live in the city centre of Montpellier, where the density and diversity of food outlets would be higher.

### Convenient grocery shopping practices

Households in the *‘Convenience’ cluster* tend to buy a large portion of their food from convenience stores or online. They are less likely to consider grocery shopping as a pleasurable activity or seek out promotions; however, they are more likely to enjoy conversing with vendors and other customers while shopping for food. In France, convenience stores are typically small and allow for more frequent interactions between customers and retailers, unlike large supermarkets where social interactions are limited^(43)^. This cluster falls somewhere between a utilitarian and a hedonic profile, where promotions are not the main interest but grocery shopping is not seen as a pleasurable activity. The households in this cluster are more likely to consist of young, single adults who have previously been associated with higher rates of online food shopping because they are more inclined to use digital tools than older adults^(44)^.

Households of the *‘Convenience’ cluster* might be characterised by a preference for convenience and therefore aim to minimise grocery shopping trips in terms of distance or frequency. This could involve opting for readily available food options from nearby convenience stores or consolidating purchases through online platforms. However, it is important to note that this particular cluster did not exhibit any significant associations with the proximity or presence of convenience stores, or any other RFE indicator. The absence of significant associations with RFE indicators could be explained by the fact that this cluster includes convenience store users as well as online food shoppers. Households belonging to this cluster are more likely to live in the city centre of Montpellier or the surrounding municipalities, rather than around the city centre (even though these differences are not statistically significant when compared with the other clusters). This suggests that these households may be exposed to either a higher density of food outlets, with convenience stores readily available around every corner (city centre), or a lower density of food outlets (surrounding municipalities), making online grocery shopping more convenient. Indeed, households residing in the neighbouring municipalities exhibited higher average proportions of online expenditures (5·88 %, sd = 14·14) compared with households living in the city centre (0·78 %, sd = 5·32) or around the city centre (3·53 %, sd = 11·49).

### Diversified grocery shopping practices

As for the two remaining clusters, *‘Diversified’* and *‘Specialized’*, results highlighted a rather clear correspondence to the hedonic profile. Households in the *‘Diversified’ cluster*, who have diversified FSS and higher expenditures for organic stores, markets, producers, and specialised food stores, enjoy purchasing food and socialising while grocery shopping, while being less interested in promotions, cheaper foods, or efficiency. Households in the *‘Specialized’ cluster*, who have a higher share of expenditures in specialised food stores, also enjoy grocery shopping and are less concerned about the availability of cheaper food. There appears to be a link between grocery shopping in different types of food outlets and enjoying the practice of food purchasing; simply visiting or trying out different types of food stores appears to be a pleasurable experience for some consumers^(40)^.

Households in the *‘Diversified’* and *‘Specialized’ clusters* are more likely to have higher incomes, be older and highly educated, although these associations were only significant for the *‘Diversified’ cluster*. Higher income has previously been associated with patronising CSA,^(29,30)^ and farmers’ markets^(28)^; moreover, higher-priced organic food is more accessible to households with higher incomes^(27)^. Education level also consistently yields similar results in many studies, suggesting that consumers with higher levels of education are more likely to purchase organic products^(45)^. The main difference between the *‘Diversified’* and *‘Specialized’ clusters* is employment status. Household heads in the *‘Diversified’* cluster are more likely to be employed than those in other clusters, while the opposite is true for the *‘Specialized’ cluster*. Households that shop in different specialist shops may have to spend more time grocery shopping, which might not always coincide with having a full or even a part-time job. Additionally, households in the *‘Specialized’ cluster* are more likely to be composed of multiple adults without children, where the adult in charge of groceries might be unemployed or retired.

Our study highlights positive associations between belonging to the *‘Diversified’ cluster* and the presence of a food market in households’ activity space, as well as the perceived availability of a producer. The presence of a market within one’s activity space may potentially encourage households to use markets. Multivariate analyses further revealed that households in the *‘Specialized’ cluster* were more likely to live close to a specialised food store and within a 500-m distance of a greengrocer. These results suggest that proximity to specialised food stores, regardless of households’ socio-economic characteristics, might encourage consumers to purchase from different specialty shops, thereby diversifying their food shopping practices. Moreover, both the *‘Diversified’* and *‘Specialized’ clusters* were more likely to be located in the city centr of Montpellier, highlighting the potential role of density and diversity of food stores in facilitating the use of diversified food shopping practices. However, due to the cross-sectional nature of this study, the possibility of reverse causality cannot be entirely ruled out. It is plausible that households with specific shopping preferences may have intentionally chosen to reside in a neighbourhood that aligns with their shopping preferences.

### Overview, future research and policy implications

While we expected strong associations between store choice and the RFE, our findings indicate that exposure to food stores may not always lead to their use. This is because exposure does not guarantee access, affordability, accommodation or acceptability^(46)^. Despite testing numerous environmental indicators, only a few were found to be significantly associated with specific grocery shopping practices, suggesting a limited but existing influence of the RFE on food store use. RFE indicators were not systematically associated with food purchasing practices, as they are shaped by economic and environmental factors, as well as individual consumer preferences and personal concerns. Studies have identified price^(47–49)^, availability and quality^(48,49)^ of food products, as well as proximity^(48,49)^, convenience^(47–49)^, cleanliness^(48,49)^, and safety^(47–49)^ of food stores as primary reasons for food store choice. Thus, RFE indicators alone cannot capture the non-geographic dimensions of the RFE^(14)^. Nevertheless, our study identified nine RFE indicators that were significantly associated with our identified clusters of food purchasing practices, independent of household characteristics. This suggests some influence of the RFE on grocery shopping practices and food store choice.

RFE indicators assessed around the home better captured associations with proximity stores (e.g. convenience stores, greengrocers and other specialised food stores), while RFE indicators assessed in households’ activity space captured associations with FSS (e.g. e-supermarkets and markets) that were missed when exposure was limited to the residential area. Additionally, perceived RFE indicators accounted for households’ interest in or knowledge of a given FSS (e.g. producers), which is overlooked with objective RFE indicators. Indeed, subjective indicators capture individuals’ perceptions and experiences of their RFE, eventually including factors like convenience, affordability, store ambiance and perceived food quality. Furthermore, objective RFE indicators are calculated within predefined buffer zones which remain uniform across households, whereas perceived RFE indicators offer greater flexibility since activity spaces are determined individually by each respondent. Both types of indicators measure the RFE in two different ways and are thus complementary. This also applies for the presence and proximity indicators. While presence indicators are calculated within predetermined buffer zones, proximity indicators, which are determined by the distances from home to the FSS, are not constrained by predefined buffer zones. Therefore, it is essential for future studies to consider both perceived and objective RFE indicators, as well as assessments around the home (proximity and presence indicators) and in activity space. Using multiple measures is important for a comprehensive understanding of how the RFE shapes food purchasing behaviours, as these diverse measures capture various facets of the FE, complementing one another in providing a comprehensive perspective. Further studies are crucial in order to deepen our understanding of the relationship between grocery shopping habits, contextual and socio-economic factors. These studies could help identify disparities in food access, inform targeted policy interventions and ultimately contribute to the promotion of healthier eating habits, the reduction of health inequities and the improvement of public health outcomes.

### Strengths and limitations

Strengths of our study include the use of objective data on households’ monthly food purchasing practices, gathered from grocery receipts over a 1-month period. This helped to mitigate the known declaration biases^(50)^, often present in self-report measures. Our study also utilised an original approach to measure households’ exposure to the FE, taking into account household members’ main places of activity and their commuting journeys, in order to calculate households’ activity space. This approach is important because consumers’ exposure to the FE is not limited to the surrounding residential areas but also includes non-residential places of activities related to social activities and travel behaviours^(8)^. The use of road network buffers to evaluate the presence of FSS around residential and activity areas constitutes a strong aspect of this study. Although Euclidean distances were employed for proximity indicators, it is important to note that in dense urban areas, Euclidean distances do not significantly differ from network distances^(51,52)^. Additionally, we studied a wide range of food shopping sites, including specialised food stores, to take into account the diversity of FSS to which French households are exposed. This is especially significant in a French urban setting, where specialised food stores such as greengrocers, bakeries and butchers are more abundant and used more frequently than in other countries^(18)^. Given that the majority of meals are eaten at home in France^(53)^, the Mont’Panier study focused exclusively on food and beverage purchases for at-home consumption, and restaurants and fast-food outlets were thus not considered in this study. However, studies conducted within different contexts might find it relevant to account for food consumption outside the home. Finally, we used both perceived and objective RFE indicators, which is important for a comprehensive understanding of the FE^(14)^.

There were some limitations to our study. First, while we assessed both the objective and perceived RFE, we did not take into account the perception of in-store availability of food products and food prices. This is an important consideration, as the affordability of food products may differ according to FSS. Second, our study was limited to a single metropolitan area, so caution is needed when extrapolating these results to the entire French population. Results may differ in less densely populated urban areas or in rural settings, although it is worth noting that the MMA is representative of most medium-sized French cities. Furthermore, it is important to note that this study did not account for neighbourhood self-selection, wherein individuals choose to reside in neighbourhoods that align with their preferred lifestyle and offer suitable facilities and resources^(54)^, raising thus the possibility of reverse causality in the studied associations. Therefore, future research should consider conducting longitudinal studies on store openings and their impact on purchases or intervention studies with appropriate designs to explore causal relationships. Another potential limitation of this study is exposure misspecification, as the utilised 500/1000-m buffers may not accurately capture the pertinent spatial scale. Indeed, previous research has indicated that grocery purchasing predominantly occurs outside the immediate neighbourhood^(55,56)^. In addition, while food purchasing practices across households were assessed over different months, potential seasonality effects were not considered in the analyses. Finally, selection bias may be an issue, as households in our study were mostly highly educated. However, we performed quota sampling based on household composition and age of household head, and all analyses presented were conducted on a weighted sample adjusted by calibration on margins based on income per unit of consumption and household composition crossed with household head’s age group to limit selection bias.

### Conclusions

This study highlights the diverse grocery shopping patterns in French households and their association with household and environmental characteristics, indicating the influence of both dimensions on food store choice. Our findings underscore the importance of considering both perceived and objective RFE indicators, as well as assessments around the home and in activity spaces, since all these measurements are complementary. Furthermore, our study shows that households with diversified FSS have higher incomes and enjoy doing their groceries more compared with under one-roof food shoppers, who have lower incomes and are more interested in promotions and finding the best prices. Potential social disparities outlined in this study underscore the utmost importance of ensuring access to both affordable and nutritious food options.

Gaining insight into the demographics of individuals using various food stores can shed light on potential social disparities and food access inequalities driven by differences in affordability that may affect diet quality. Future research delving into the variations in purchasing behaviour across different types of stores and their associations with the nutritional quality of food purchases can build on the insights gained from this research. Research on the factors that drive food store choices is crucial and should be conducted in diverse geographical settings, considering consumers’ food culture, which is deeply linked to their food shopping practices^(16,17)^. In France, the geographical setting is very different from that of the USA, where the habitat is more dispersed and where geographical accessibility might play a greater role in food outlet use.

Understanding grocery shopping practices and associated RFE characteristics is important, especially in the context of the recent COVID-19 pandemic, which has raised concerns about food access^(57,58)^. The pandemic has led to substantial disruptions in various aspects of daily life, including shifts in food shopping behaviours. While there has been a substantial increase in online grocery shopping, traditional supermarkets have maintained their enduring importance^(57)^. This study offers a valuable pre-pandemic baseline, providing essential context for research investigating how purchasing patterns have changed during and after the pandemic.

Research on where people buy their foods and how they engage with their FE can help in the decision-making process concerning food policies designed to improve the FE. Future research should examine the social dynamics that influence grocery shopping practices and take into account the in-store availability of food products, food prices and consumer preferences. Such studies would help understand how grocery shopping practices are shaped and might help improve the FE and consequently people’s health^(47)^.

## Supporting information

Recchia et al. supplementary material 1 Recchia et al. supplementary material

Recchia et al. supplementary material 2 Recchia et al. supplementary material

## Supplementary material

For supplementary material accompanying this paper visit https://doi.org/10.1017/S1368980023002720
