# Supplementary material for: Store-specific grocery shopping patterns and their association with objective and perceived retail food environments
Source: Public Health Nutr. 2023 Dec 11;27(1):e13. doi: 10.1017/S1368980023002720 (PMC10830372; doi:10.1017/S1368980023002720)
Supplement: Recchia et al. supplementary material 1 — Recchia et al. supplementary material [file S1368980023002720sup001.docx]

**Additional file 1.** Results of the Principal Component Analysis

|  | **Eigenvalue** | **Percentage  of variance** | **Cumulative percentage  of variance** |
| --- | --- | --- | --- |
| **comp 1** | 1.73 | 15.76 | 15.76 |
| **comp 2** | 1.33 | 12.08 | 27.85 |
| **comp 3** | 1.25 | 11.38 | 39.23 |
| **comp 4** | 1.13 | 10.31 | 49.53 |
| **comp 5** | 1.08 | 9.79 | 59.32 |
| **comp 6** | 1.00 | 9.09 | 68.41 |
| **comp 7** | 0.94 | 8.53 | 76.93 |
| **comp 8** | 0.92 | 8.34 | 85.27 |
| **comp 9** | 0.84 | 7.64 | 92.91 |
| **comp 10** | 0.77 | 7.04 | 99.96 |
| **comp 11** | 0.00 | 0.04 | 100.00 |
