# Supplementary material for: Store-specific grocery shopping patterns and their association with objective and perceived retail food environments
Source: Public Health Nutr. 2023 Dec 11;27(1):e13. doi: 10.1017/S1368980023002720 (PMC10830372; doi:10.1017/S1368980023002720)
Supplement: Recchia et al. supplementary material 2 — Recchia et al. supplementary material [file S1368980023002720sup002.docx]

**Additional file 2.** Buyer profile, satisfaction with the food environment and wished improvements concerning the food environment

|  | **Total**  **sample** | **Cluster**  **Supermarket**  **49%** | | **Cluster**  **Diversified**  **18%** | | **Cluster**  **Discount**  **12%** | | **Cluster**  **Convenience**  **12%** | | **Cluster**  **Specialized**  **9%** | |
| --- | --- | --- | --- | --- | --- | --- | --- | --- | --- | --- | --- |
|  | **%^1^** | **%^1^** | **p*^2^*** | **%^1^** | **p*^2^*** | **%^1^** | **p*^2^*** | **%^1^** | **p*^2^*** | **%^1^** | **p*^2^*** |
| **Buyer profile** |  |  |  |  |  |  |  |  |  |  |  |
| Efficacy | 59% | 61% | 0.5 | 45% | **0.01** | 67% | 0.3 | 64% | 0.6 | 61% | 0.8 |
| Best prices | 56% | **69%** | **<0.001** | 24% | **<0.001** | **73%** | **0.02** | 42% | **0.05** | 49% | 0.4 |
| Pleasure | 53% | 49% | 0.1 | **64%** | **0.03** | 54% | >0.9 | 40% | **0.09** | **68%** | **0.07** |
| Social interactions | 45% | 36% | **0.002** | **61%** | **0.004** | 32% | **0.06** | **61%** | **0.03** | 58% | 0.1 |
| Meal preparation | 84% | 86% | 0.6 | 81% | 0.4 | 79% | 0.4 | 86% | 0.8 | 90% | 0.3 |
| **Satisfaction with the food environment** | 86% | 89% | 0.1 | 88% | 0.6 | 85% | 0.7 | 78% | 0.1 | 82% | 0.4 |
| **Improvements concerning the availability of** |  |  |  |  |  |  |  |  |  |  |  |
| Supermarkets | 5% | 6% | 0.3 | 1.4% | 0.1 | 9% | 0.2 | 0% | 0.1 | 6% | 0.8 |
| Cheaper food | 26% | **34%** | **0.003** | 8% | **<0.001** | **42%** | **0.02** | 22% | 0.5 | 8% | **0.02** |
| Markets | 34% | 35% | 0.8 | 33% | 0.9 | 31% | 0.6 | 32% | 0.8 | 40% | 0.5 |
| Proximity stores | 21% | 18% | 0.2 | 25% | 0.3 | 15% | 0.3 | 25% | 0.6 | **33%** | **0.09** |
| Online food shopping | 10% | 11% | 0.4 | 2% | **0.02** | 15% | 0.2 | 10% | >0.9 | 13% | 0.6 |

^1^ weighted percentage; ^2^ chi-squared test with Rao & Scott's second-order correction. The sample was adjusted by calibration on margins based on income per unit of consumption and household composition crossed with household head’s age group. The numbers in bold represent percentages higher than those of the total sample, and p-values < 0.1.
